# The significance of TNF‐α and MMP‐8 concentrations in non‐invasively obtained amniotic fluid predicting fetal inflammatory response syndrome

**DOI:** 10.1002/ijgo.14478

**Published:** 2022-10-07

**Authors:** Violeta Gulbiniene, Greta Balciuniene, Irena Dumalakiene, Rita Viliene, Ingrida Pilypiene, Diana Ramasauskaite

**Affiliations:** ^1^ Center of Obstetrics and Gynaecology, Institute of Clinical Medicine of the Faculty of Medicine Vilnius University Vilnius Lithuania; ^2^ State Research Institute Centre for Innovative Medicine Vilnius Lithuania

**Keywords:** amniotic fluid, biomarker, fetal inflammatory response syndrome (FIRS), matrix metalloproteinase‐8 (MMP‐8), non‐invasive method, preterm birth, preterm premature rupture of membranes (PPROM), tumor necrosis factor‐α (TNF‐α)

## Abstract

**Objective:**

To determine the significance of tumor necrosis factor‐α (TNF‐α) and matrix metalloproteinase‐8 (MMP‐8) in vaginally obtained amniotic fluid predicting fetal inflammatory response syndrome (FIRS) after preterm premature rupture of membranes (PPROM).

**Methods:**

In this prospective case–control study, TNF‐α and MMP‐8 concentrations were evaluated in vaginally obtained amniotic fluid from women with PPROM at 22–34 weeks of pregnancy. Biomarkers' concentrations were determined using an enzyme‐linked immunosorbent assay. Patients were divided into two groups: the FIRS group (cord blood interleukin‐6 > 11 pg/ml or histological funisitis) and the non‐FIRS group (without these findings). The data were analyzed using R package (R–4.0.5).

**Results:**

The median TNF‐α and MMP‐8 concentrations in amniotic fluid from the 145 women included in the study were higher in the FIRS group than in the non‐FIRS group. The area under the curve of TNF‐α and MMP‐8 was 0.77 and 0.75, respectively. The TNF‐α concentration cut‐off predicting FIRS was 89.20 pg/ml and was 170.76 pg/ml for MMP‐8. In regression analysis, MMP‐8 concentration was an independent predictor for FIRS. An MMP‐8 concentration greater than 170 ng/ml and a TNF‐α concentration greater than 89 pg/ml increased the odds of FIRS 7.62 and 14.92 times, respectively.

**Conclusions:**

MMP‐8 and TNF‐α concentrations in vaginally obtained amniotic fluid may be good predictors for FIRS after PPROM before 34 weeks of pregnancy. The non‐invasive amniotic fluid analysis could be an alternative method to invasive amniocentesis.

## Synopsis

MMP‐8 and TNF‐α in vaginally obtained amniotic fluid are significant predictors for FIRS after PPROM; non‐invasive analysis could be an alternative to invasive amniocentesis.

## 1 INTRODUCTION

Fetal inflammatory response syndrome (FIRS) is a systemic inflammatory response of the fetus, with an increase in the range of cytokines due to intra‐amniotic infection and/or inflammation.^1^ FIRS was originally observed in fetuses with preterm birth and preterm premature rupture of membranes (PPROM).^2^ This syndrome is determined by an elevated fetal plasma interleukin‐6 (IL‐6) level above 11 pg/ml^2^ or identification of histological funisitis.^3^ FIRS leads to increased neonatal morbidity and mortality, with short‐ and long‐term outcomes: respiratory distress syndrome, sepsis, bronchopulmonary dysplasia, intraventricular hemorrhage, periventricular leukomalacia, retinopathy of prematurity, sensorineural hearing loss and neurodevelopmental disabilities, including cerebral palsy.^1^, ^2^, ^4^, ^5^, ^6^

Tumor necrosis factor‐α (TNF‐α) is a cytokine that plays a key role in the onset of the inflammatory cascade and mediating septic shock and death.^7^ It is a multifunctional diverse factor that induces cell survival and death, activates and suppresses angiogenesis, recruits and regulates immune cells, and assists in constructing the extracellular matrix (ECM).^8^ TNF‐α demonstrates a dose‐related effect on cells, causing induction of the processes at low levels and suppression or destruction at high levels.^8^ During inflammation and/or infection in the amniotic cavity, TNF‐α is found in high concentrations in amniotic fluid and suppresses the growth of amnion cells, stimulates the prostaglandin synthesis, and induces the release of matrix metalloproteinases (MMPs), thus triggering PPROM and preterm delivery.^9^

Matrix metalloproteinase‐8 (MMP‐8) (also referred to as neutrophil collagenase and collagenase 2), is an ECM‐degrading enzyme that belongs to the MMP family and is released from activated neutrophils.^10^ MMP‐8 has a significant role at the site of inflammation, initiating the breakdown of ECM, mediating tissue remodeling, angiogenesis, and wound healing.^10^, ^11^ During intra‐amniotic bacterial invasion, this enzyme is responsible for the degradation of ECM of the chorioamnion, resulting in premature rupture of membranes (PROM), preterm birth, and is found in high levels in the amniotic fluid as well.^11^

Amniotic fluid TNF‐α and MMP‐8 are both associated with intra‐amniotic infection/inflammation,^11^, ^12^, ^13^, ^14^, ^15^ FIRS,^16^, ^17^ and adverse neonatal outcomes.^16^, ^18^, ^19^, ^20^, ^21^ Although various amniotic fluid biomarkers have been extensively researched as predictive tools for intra‐amniotic inflammation/infection and FIRS, most studies were sampling amniotic fluid obtained by transabdominal amniocentesis.^2^, ^11^, ^13^, ^14^, ^17^, ^18^, ^20^ Only a few studies have investigated non‐invasively obtained amniotic fluid to predict FIRS,^16^, ^22^, ^23^ and even fewer have evaluated the role of MMP‐8 and TNF‐α in vaginally obtained amniotic fluid prognosticating FIRS.^16^, ^22^

Here we aimed to determine the significance of TNF‐α and MMP‐8 concentrations in vaginally obtained amniotic fluid predicting FIRS in patients with PPROM before 34 weeks of pregnancy.

## 2 MATERIALS AND METHODS

**FIGURE 1:**
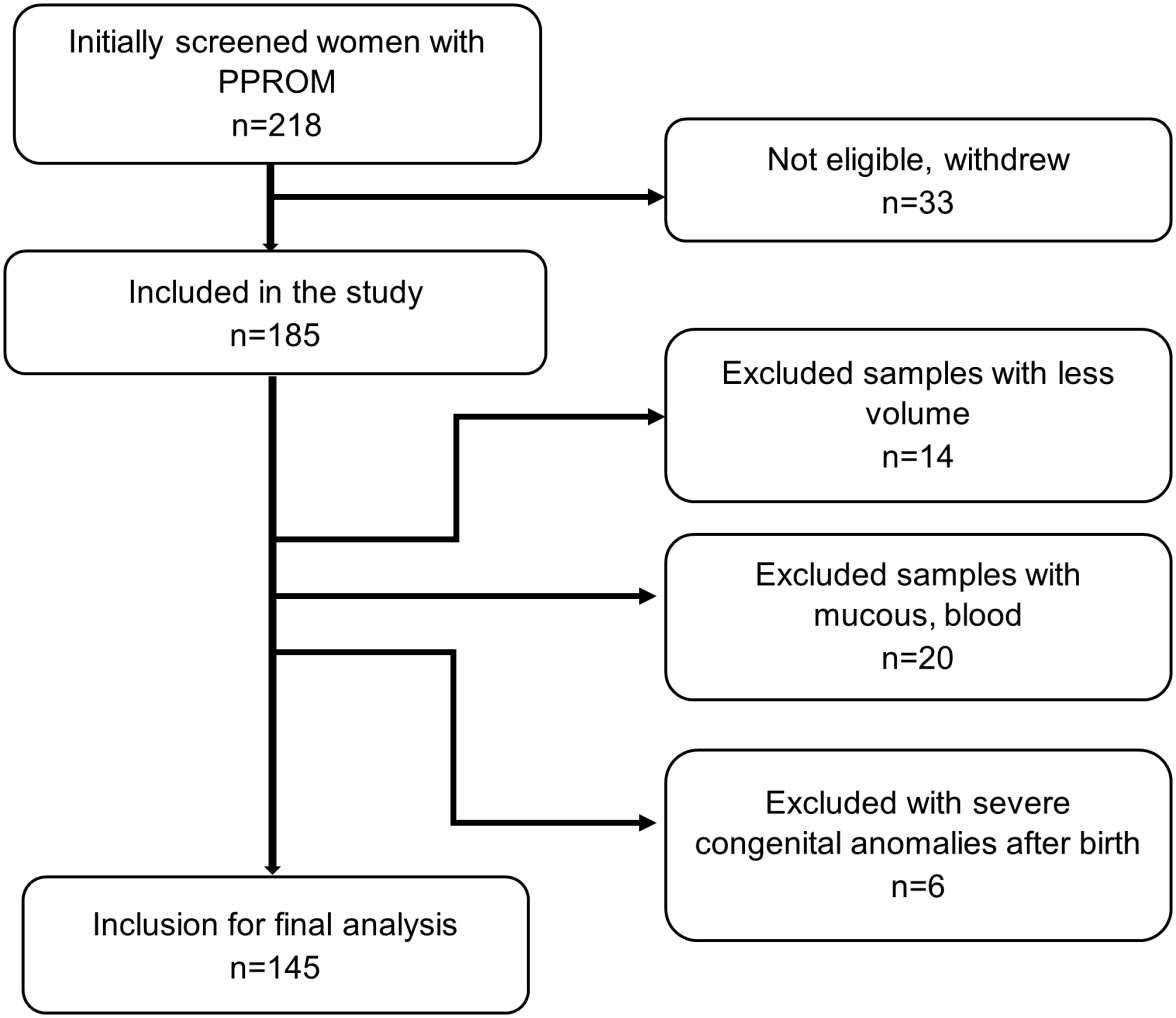
Subject selection flowchart.

A prospective case–control study was performed in Vilnius University Hospital Santaros Klinikos and funded by the Research Council of Lithuania under grant no. S–MIP–19–57. We assessed patients with PPROM hospitalized between 2017 and 2020 (Figure 1). The study included 185 singleton pregnant women with PPROM at 22–34 weeks of pregnancy. Exclusion criteria were multiple gestations, vaginal bleeding, placenta previa, fetal and neonatal malformations, and non‐reassuring fetal status. Additionally, we excluded 34 patients due to inadequate specimens (with mucus, blood, lower volume) and six cases due to severe congenital anomalies diagnosed postnatally. The final analysis included 145 participants. All patients provided informed written consent. The Vilnius Regional Biomedical Research Ethics Committee approved our research protocol (no. 158200–17–931–434).

Gestational age was based on the last menstrual period and confirmed or modified by an ultrasound scan at 11^+0^–13^+6^ weeks of gestation. PROM was identified by examination with a sterile speculum to verify the pooling of amniotic fluid in the vagina or from the cervix. In doubtful cases, PROM was validated by the presence of the placental alpha microglobulin‐1 protein in the cervicovaginal fluid (Qiagen).

Free leaking amniotic fluid was collected vaginally with a sterile centrifuge tube every 2 days. The sample obtained less than 48 h before labor was included in further analysis. We chose an interval of less than 48 h between amniotic fluid sampling and labor as the levels of markers may change during a long sampling period due to the increased risk of intra‐amniotic infection after a long latency period. Furthermore, the closer the period to delivery, the less likely the value of amniotic fluid markers could change, and the more they reflect the predicted effect in the newborn.

Minimizing contamination and attaining clear specimens, samples were centrifuged at 3000 rpm for 5 min at 4°C and stored at −80°C. Immunological amniotic fluid assays of TNF‐α and MMP‐8 were performed using an enzyme‐linked immunosorbent assay (ELISA) with commercial ELISA kits (Bender MedSystems). For the ELISA, non‐diluted specimens were used to determine TNF‐α concentrations. If measured concentrations of analytes exceeded the highest point on the standard curve, we performed dilutions of 1:2, 1:5, or 1:10. Samples for the analysis of MMP‐8 were diluted to 1:10. Diluents were provided by the manufacturer. The concentrations of cytokines were calculated according to standard curves using a special program for the evaluation of ELISA results: Gen5 Microplate Data Collection & Analysis Software (BioTek Instruments).

FIRS was defined according to the umbilical cord blood IL‐6 levels greater than 11 pg/ml and/or histological funisitis.^2^, ^3^ After birth, umbilical cord serum samples were collected and the IL‐6 concentration was determined by automated chemiluminescent enzyme immunoassay using a kit (DPC). Funisitis was diagnosed by the hospital pathologist if neutrophilic infiltration of the umbilical vessels or Wharton's gel was detected on histological examination of the umbilical cord. Researchers were blinded to biomarker levels.

According to the hospital protocol, patients with PPROM before 34 weeks of pregnancy were on expectant management with antibiotics, one course of prenatal corticosteroids, and, if needed, tocolytics during the lung maturation course. Antibiotic therapy included intravenous ampicillin (2 g every 6 h) and erythromycin (250 mg every 6 h) for 48 h followed by oral amoxicillin (500 mg every 8 h) and erythromycin (250 mg every 6 h) for 5 days additionally. Two 12‐mg doses of dexamethasone were administered intramuscularly every 12 h to accelerate fetal lung maturation. The spontaneous beginning of labor or labor induction followed after fetal lung maturation. Indications for labor induction were intrauterine infection according to Gibb's criteria, hemorrhage, or compromised fetal condition. Participation in the study did not change routine clinical practice.

A statistical analysis was performed with R software version R–4.0.5. (R Core Team [2021]). The Shapiro–Wilk test determined the distribution of the data. Baseline differences between groups were determined using the Student *t*‐test, Mann–Whitney‐Wilcoxon test, Kruskal‐Wallis test, χ^2^ test, or Fisher exact test as appropriate. We present data as mean with standard deviation or median and interquartile range for continuous variables and frequencies and percentages for categorical variables. We used the receiver operating characteristic (ROC) curve method to evaluate the ability of variables to discriminate between groups and the DeLong method to compare the areas under the curves (AUC) of different models. The Youden index determined the best cut‐off values to predict FIRS. We applied the multiple regression analysis to estimate the reliability of biomarkers predicting FIRS and compared these models with ANOVA; standard errors and odds ratios are reported with confidence intervals. A *P* value of less than 0.05 was considered statistically significant.

## 3 RESULTS

We included a total of 145 women and their neonates in the final analysis. The study population was grouped into the FIRS group (*n* = 54) and the non‐FIRS group (*n* = 91) based on FIRS diagnosis. Table 1 shows the demographic and clinical characteristics of the study population. Most maternal factors did not differ between groups, except hypertensive disorders were diagnosed more often in the non‐FIRS group and histological chorioamnionitis was more common in the FIRS group. Infants with FIRS had a lower birth weight, lower gestational age, and more often had respiratory distress and Apgar scores less than seven at 1 and 5 min after birth than neonates without FIRS. The rate of sepsis, bronchopulmonary dysplasia, neonatal death, and umbilical cord arterial pH did not differ.

**TABLE 1:** Demographic and clinical characteristics of the study population

	Total cohort (*n* = 145)	FIRS group (*n* = 54)	Non‐FIRS group (*n* = 91)	*P* value
Maternal characteristics				
Age of mother (year)	31.25 ± 5.62	31.22 ± 5.5	31.26 ± 5.7	0.966
Latency period (h)	17.7 (6.2–51)	15.9 (4.0–50)	18.9 (8.5–54.1)	0.409
Hypertensive disorders	31 (21%)	6 (11%)	25 (28%)	**0.020**
Gestational diabetes	34 (24%)	12 (22%)	22 (24%)	0.761
Gestational anemia	38 (26%)	16 (30%)	22 (24%)	0.470
GBS positive	17 (12%)	7 (13%)	10 (11%)	0.444
Gravidity				
Primigravida	50 (34%)	20 (37%)	30 (33%)	0.618
Multigravida	95 (66%)	34 (63%)	61 (67%)	
Parity				
Primiparous	68 (47%)	23 (43%)	45 (50%)	0.424
Multiparous	77 (53%)	31 (57%)	46 (51%)	
Histological chorioamnionitis	54 (37%)	38 (70%)	16 (18%)	**<0.001**
Funisitis	21 (14%)	21 (39%)	0	**<0.001**
Neonatal characteristics				
Gestational age at birth (week)	32 (30–34)	31.5 (28–33)	33.0 (31–34)	**0.003**
Birth weight (g)	1840 ± 634	1675 ± 640	1938 ± 613	**0.020**
Apgar scores <7 at 1 min	22 (15%)	14 (26%)	8 (9%)	**0.005**
Apgar scores <7 at 5 min	7 (25%)	6 (11%)	1 (1%)	**0.007**
Umbilical cord arterial pH	7.34 (7.28–7.39)	7.35 (7.28–7.41)	7.33 (7.28–7.39)	0.292
RDS	122 (84%)	51 (94%)	71 (78%)	**0.009**
Neonatal death	1 (1%)	1 (2%)	0	0.193
Sepsis	15 (10%)	5 (9%)	10 (11%)	0.741
BPD	17 (12%)	9 (17%)	8 (9%)	0.142

*Note*: The data are presented as median (interquartile range) or mean with standard deviation for continuous variables and as number (percent) for categorical variables. Significant *P* values are in bold. Abbreviations: BPD, bronchopulmonary dysplasia; FIRS, fetal inflammatory response syndrome; GBS, a group B streptococcus test; RDS, respiratory distress.

**FIGURE 2:**
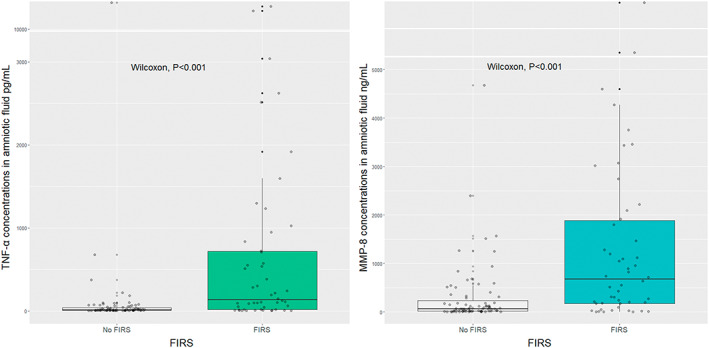
Median concentrations of tumor necrosis factor‐α (TNF‐α) and matrix metalloproteinase‐8 (MMP‐8) in amniotic fluid in the fetal inflammatory response syndrome (FIRS) and the non‐FIRS groups (TNF‐α: 136.43 pg/ml [interquartile range 17.65–718.59] vs. 14.35 pg/ml [interquartile range 6.71–39.58], *P* < 0.001; MMP‐8: 673.30 ng/ml [interquartile range 175.02–1886.61] vs. 65.57 ng/ml [interquartile range 15.69–234.54], *P* < 0.001).

The median MMP‐8 and TNF‐α concentrations were 10‐fold higher in the FIRS group than in the non‐FIRS group; the difference was significant (*P* < 0.001) (Figure 2).

**FIGURE 3:**
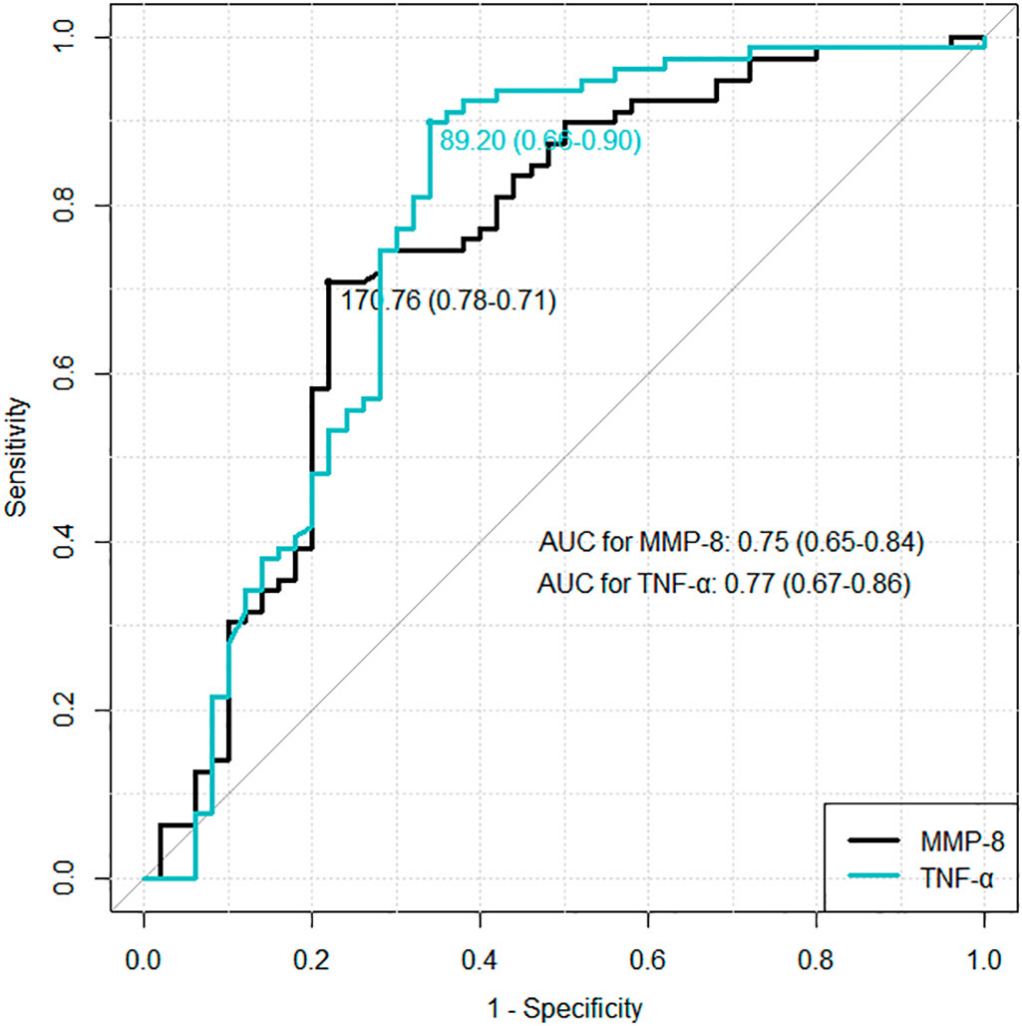
Comparison of the receiver operating characteristic (ROC) curves of matrix metalloproteinase‐8 (MMP‐8) and tumor necrosis factor‐α (TNF‐α). AUC, area under the curve.

We constructed ROC curves for MMP‐8 and TNF‐α levels in amniotic fluid to distinguish neonates with and without FIRS (MMP‐8: AUC, 0.75; confidence interval 0.65–0.84; TNF‐α: AUC, 0.77, confidence interval 0.67–0.86) (Figure 3).

AUC of TNF‐α and MMP‐8 did not differ with the DeLong test (*P* = 0.507). Using ROC curves, we selected cut‐off values for amniotic fluid biomarkers: 89.20 pg/ml for TNF‐α concentration and 170.76 ng/ml for MMP‐8 level. Table 2 shows the diagnostic parameters of amniotic fluid MMP‐8 and TNF‐α concentrations to detect FIRS.

**TABLE 2:** Diagnostic parameters of amniotic fluid biomarkers for the detection of fetal inflammatory response syndrome

Amniotic fluid marker	Sensitivity (%)	Specificity (%)	Positive prognostic value (%)	Negative prognostic value (%)
TNF‐α > 89.20 pg/ml	66	90	80	81
MMP‐8 > 170.76 ng/ml	78	71	63	84

Abbreviations: MMP‐8, matrix metalloproteinase‐8; TNF‐α, tumor necrosis factor‐α.

We performed regression analysis assessing the significance of TNF‐α and MMP‐8 to predict FIRS after adjustment for gestational age (Table 3). The analysis revealed that MMP‐8 significantly predicted FIRS. The input variable of TNF‐α was not significant. After adding both markers in one model, MMP‐8 remained a significant predictor, whereas TNF‐α was not. The combined model of both biomarkers was not superior to the MMP‐8 model. Altogether, MMP‐8 was a significant independent predictor for FIRS in non‐invasively obtained amniotic fluid.

**TABLE 3:** The regression analysis predicting fetal inflammatory response syndrome (FIRS) with matrix metalloproteinase‐8 (MMP‐8) and tumor necrosis factor‐α (TNF‐α) with and without cut‐off values, with adjustment for gestational age: FIRS as outcome variable; MMP‐8, TNF‐α, MMP‐8 cut‐off > 170 ng/ml (MMP‐8 > 170) and TNF‐α cut‐off > 89 pg/ml (TNF‐α > 89) as input variables

Models	ANOVA *P* value	Coefficient	β	Standard error	*P* value	Adjusted odds ratio	Confidence interval
Models with total dataset							
MMP‐8 + TNF‐α	**<0.001** ^a^	MMP‐8	0.0008	0.0003	**0.002**	NA	NA
	0.256^b^	TNF‐α	0.0001	0.0001	0.312	NA	NA
TNF‐α	**<0.001** ^a^	TNF‐α	0.0002	0.0001	0.209	NA	NA
MMP‐8	0.256^b^	MMP‐8	0.0009	0.0003	**0.002**	NA	NA
Models with cut‐off values							
MMP‐8 > 170 + TNF‐α > 89	<0.001^c^	MMP‐8 > 170	0.9696	0.53	0.069	2.64	0.90–7.51
	0.075^d^	TNF‐α > 89	2.0975	0.58	**<0.001**	8.15	2.69–27.02
TNF‐α > 89	0.075^d^	TNF‐α	2.7030	0.49	**<0.001**	14.92	5.91–41.79
MMP‐8 > 170	**<0.001** ^c^	MMP‐8 > 170	2.0310	0.43	**<0.001**	7.62	3.37–18.32

*Note*: Significant results are in bold.

^a^ Comparing TNF‐α model with MMP‐8 + TNF‐α model using ANOVA.

^b^ Comparing MMP‐8 model with MMP‐8 + TNF‐α model using ANOVA.

^c^ Comparing MMP‐8 > 170 + TNF‐α > 89 model with MMP‐8 > 170 model using ANOVA.

^d^ Comparing TNF‐α > 89 model with MMP‐8 > 170 + TNF‐α > 89 model using ANOVA.

Due to the dose‐dependent effect of TNF‐α, we tested predictive models adjusting for gestational age with optimal cut‐off values (Table 3). An MMP‐8 level greater than 170 ng/ml increased the odds of FIRS 7.62 times. The odds for FIRS were 14.92 times higher with a TNF‐α level greater than 89 pg/ml. In the combined model, holding gestational age and MMP‐8 at fixed values, TNF‐α significantly increased the odds of FIRS. Conversely, an MMP‐8 level greater than 170 ng/ml was insignificant in the combined model, controlling gestational age and TNF‐α. Comparing single models with the combined one, the ANOVA test revealed that the combined model is superior to the model of MMP‐8 greater than 170 ng/ml, but not superior to the model of TNF‐α greater than 89 pg/ml. In general, both amniotic fluid biomarkers were significant predictors of FIRS in multivariate regression with cut‐off values adjusting for gestational age. In the combined model, a TNF‐α cut‐off of greater than 89 pg/ml was a superior predictor of having FIRS than MMP‐8 level.

## 4 DISCUSSION

In this study, we researched whether TNF‐α and MMP‐8 concentrations in non‐invasively obtained amniotic fluid could serve as predictive markers for FIRS in patients after PPROM before 34 weeks of pregnancy.

Our study used the non‐invasive sampling technique for amniotic fluid analysis to its best advantage. Previous studies on amniotic fluid biomarkers obtained samples predominantly by amniocentesis.^2^, ^11^, ^13^, ^14^, ^17^, ^18^, ^20^ Only a few studies have published results of a cervicovaginal fluid analysis to detect FIRS.^16^, ^22^, ^23^, ^24^ We reinforce the concept of the non‐invasive amniotic fluid analysis as an alternative to amniocentesis predicting FIRS after PPROM. Vaginally obtained amniotic fluid TNF‐α and MMP‐8 concentrations were strong predictors for FIRS. Ultimately, cut‐off values for both markers were determined.

Thus far, several studies have reported an association between TNF‐α and MMP‐8 concentrations and FIRS^16^, ^17^ and these findings are consistent with our results. We found 10‐fold higher medians of TNF‐α and MMP‐8 concentrations in the FIRS group than in the non‐FIRS group. Park et al.^17^ revealed that a nine‐fold higher median concentration of MMP‐8 in amniotic fluid was associated with funisitis in patients with histologic chorioamnionitis. Correspondingly, Kunze et al.^16^ determined that median TNF‐α levels in vaginal amniotic fluid differed 10‐fold between the FIRS group and controls, and TNF‐α improved AUC of the clinical predictive model. It also accords with our observations that, based on ROC curves and AUC, TNF‐α and MMP‐8 are good discriminators in neonates with and without FIRS, and the performance of both markers was similar.

In addition, cut‐off values for amniotic fluid biomarkers to identify FIRS were determined: 89.20 pg/ml for TNF‐α and 170.76 ng/ml for MMP‐8. Both biomarkers showed high performance in terms of quality and value of tests. Kunze et al.^16^ noted cut‐offs for TNF‐α of 200 pg/ml and 300 pg/ml with different immunoassays in vaginal secretions. Park et al.^17^ stated that an MMP‐8 cut‐off of 23 ng/ml in amniotic fluid obtained by transabdominal amniocentesis indicated funisitis best. There is a scarcity of research on MMP‐8 concentration in vaginal amniotic fluid, and, to the best of our knowledge, cut‐offs for MMP‐8 concentration in vaginal amniotic fluid have not been previously reported. Dorfeuille et al.^21^ analyzed MMP‐8 in the vaginal fluid after PPROM and found an association between MMP‐8 and chorioamnionitis and adverse neonatal neurologic outcome, but FIRS was not the study's subject. Reported cut‐off values may vary due to different sampling techniques, timing, and immunoassays performed, making a direct comparison difficult. Further studies are needed to determine the best non‐invasive sampling technique and to optimize the time of specimen collection.

Interestingly, we noted that the MMP‐8 cut‐off for FIRS was similar to that for histologic chorioamnionitis (MMP‐8: 170.76 vs. 172.53 ng/ml, respectively), as reported previously,^12^ although the TNF‐α cut‐off was four‐fold higher for FIRS than for histologic chorioamnionitis (TNF‐α: 89.20 vs. 21.17 pg/ml, respectively). Based on the most common ascending route of intra‐amniotic infection,^5^ starting in the vagina/cervix, proceeding through membranes into the amniotic cavity, and then to the fetus, we expected the increase in levels of biomarkers between histologic chorioamnionitis and FIRS. Interpreting our results, we speculate that these biomarkers may signify different inflammation process aspects. MMP‐8, released by neutrophils, that infiltrate membranes and umbilicus, represents a histological feature of inflammation.^10^ TNF‐α, produced by activated macrophages, lymphoid cells, mast cells, and non‐immune cells such as endothelial cells, fibroblasts, and smooth muscle cells, expresses a biochemical measure of inflammation.^8^ That is consistent with Park et al. findings^17^ of MMP‐8 association with funisitis as the histological equivalent of FIRS, and Romero et al.^7^ and Maymon et al.^14^ results concerning TNF‐α and its soluble receptors' role in biochemical homeostasis in intra‐amniotic inflammation and FIRS.

Furthermore, MMP‐8 in vaginal amniotic fluid was an independent predictor for FIRS, even controlling for gestational age in regression analysis. These data correspond with a known fact that the best predictors of neonatal outcomes in preterm delivery with intact membranes and PPROM seem to be amniotic fluid levels of MMP‐8 and IL‐6.^15^ Unexpectedly, TNF‐α did not reveal statistical significance in general models. Considering the dose‐related TNF‐α effect,^8^ we investigated models with cut‐off values. The increase in odds for FIRS was significant: 7.62 times with MMP‐8 concentration greater than 170 ng/ml and 14.92 with TNF‐α concentration greater than 89 pg/ml. Overall, both amniotic fluid biomarkers were significant predictors of FIRS in logistic regression with cut‐off values adjusting for gestational age.

It is important to note that not all studies confirm these findings. Mikolajczyk et al.^22^ found no association between TNF‐α concentrations in cervicovaginal fluid after PPROM and umbilical cord blood. In Kayem et al. study,^24^ the relationship between vaginal TNF‐α and maternal–fetal infections was weak. In both studies, the authors collected vaginal samples at weekly intervals until delivery, which possibly affected the levels of cytokines. Furthermore, the primary outcomes of the mentioned studies differed, so it is difficult to compare results.

Our study's strengths include the analysis of non‐invasively obtained amniotic fluid, the use of FIRS as the outcome, defined by proven biochemical, histologic criteria, and patients' treatment not affected by amniotic fluid biomarkers levels. In addition, the number of patients in this study was larger than most that have examined non‐invasive amniotic fluid. We acknowledge limitations in our study, as follows: a smaller number of newborns with FIRS than controls, statistically different gestational age in the groups, a lack of standard non‐invasive amniotic fluid sampling technique, and results on the non‐invasive method not validated simultaneously with the standard approach, such as amniocentesis. As FIRS is associated with the duration of gestation,^5^ a lower gestational age was anticipated in the FIRS group. To minimize the gestational age effect, we adjusted predictive models for gestational age. Nevertheless, replication of the study in a homogeneous group of preterm patients with FIRS matching in gestational age would be beneficial. As there is no standardized non‐invasive amniotic fluid sampling technique, and each previous research used a different method,^16^, ^21^, ^22^, ^23^, ^24^ the results are not always comparable. Furthermore, regarding validation with amniocentesis, Musilova et al.^23^ previously reported a strong correlation between biomarker levels in amniotic fluid collected via amniocentesis or vaginally. Based on that, we assumed that biomarker levels in vaginal amniotic fluid reflect biomarker levels in amniotic fluid obtained by amniocentesis.

Until now, FIRS was mostly determined after birth or prenatally with invasive methods. Therefore, a sensitive non‐invasive test for the early prediction of FIRS is needed. Overall, we support the use of TNF‐α and MMP‐8 in non‐invasively obtained amniotic fluid to identify FIRS after PPROM. The evaluation of vaginally obtained amniotic fluid has proven to be non‐invasive, easily performed, informative, and without complications. The analysis of MMP‐8 and TNF‐α may improve the prediction of FIRS before birth, allowing neonatal risk to be determined earlier, and may impact the management strategy for both women with PPROM and neonates.

## AUTHOR CONTRIBUTIONS

IP, ID, and DR were responsible for conceptualization. GB, ID, and RV were responsible for methodology. GB and VG carried out the formal analysis. GB, RV, and VG carried out the investigation; ID and RV were responsible for resources. GB and VG were responsible for data curation; VG prepared the original draft of the manuscript. IP, ID, and DR reviewed and edited the manuscript. VG was responsible for visualization. IP and DR were responsible for supervision. DR was responsible for project administration and funding acquisition. All authors have read and agreed to the published version of the manuscript.

## FUNDING INFORMATION

The funders had no role in the design of the study, in the collection, analyses, or interpretation of data, in the writing of the manuscript, or in the decision to publish the results.

## CONFLICTS OF INTEREST

The authors have no conflicts of interest.

## Data Availability

The data that support the findings of this study are available from the corresponding author upon reasonable request.
